# The impact of lymphovascular invasion in patients with prostate cancer following radical prostatectomy and its association with their clinicopathological features: An updated PRISMA-compliant systematic review and meta-analysis

**DOI:** 10.1097/MD.0000000000013537

**Published:** 2018-12-10

**Authors:** Wei Jiang, Lijin Zhang, Bin Wu, Zhenlei Zha, Hu Zhao, Yuan Jun, Yuefang Jiang

**Affiliations:** a Department of Urology, Taizhou People's Hospital, The Fifth Affiliated Hospital of Medical School of Nantong University, Taizhou; b Department of Urology, Affiliated Jiang-yin Hospital of the Southeast University Medical College, Jiang-yin, Jiangsu Province, China.

**Keywords:** biochemical recurrence, lymphovascular invasion, meta-analysis., prostate cancer, radical prostatectomy

## Abstract

**Objective:**

Numerous studies have shown that lymphovascular invasion (LVI) is linked to biochemical recurrence (BCR) in prostate cancer (PCa) patients following radical prostatectomy (RP). However, the actual clinicopathological impacts of LVI remain unclear. Thus, we performed a meta-analysis to evaluate the pathologic and prognostic impacts of LVI in PCa patients.

**Methods:**

Following the guidance of the PRISMA statement, relevant studies were collected systematically from the PubMed, EMBASE, and Web of Science databases to identify relevant studies published before June 2018. The hazard ratios (HRs) and 95% confidence intervals (95% CIs) were calculated to investigate the association of LVI with BCR and clinicopathological features.

**Results:**

A total of 20 studies including 25,570 patients (106–6678 per study) with PCa were incorporated into this meta-analysis. Overall pooled analysis suggested that LVI was associated with a higher BCR risk both in univariate (pooled HR=1.50, 95% CI: 1.34–1.68, *P* <.001) and multivariate analyses (pooled HR=1.25, 95% CI: 1.17–1.34, *P* <.001). In addition, LVI was closely correlated with extraprostatic extension (yes vs no: OR = 4.23, 95% CI: 1.86–9.61, *P* <.001), pathological GS (≥7 vs <7: OR = 5.46, 95% CI: 2.25–13.27, *P* <.001), lymph node metastases (yes vs no: OR = 18.56, 95% CI: 7.82–44.06, *P* <.001), higher pathological stage (≥ T3 vs < T2: OR = 6.75, 95% CI: 5.46–8.36, *P* <.001), positive surgical margin (positive vs negative: OR = 2.42, 95% CI: 1.57–3.72, *P* <.001) and seminal vesicle invasion (yes vs no: OR = 5.72, 95% CI: 2.45–13.36, *P* <.001).

**Conclusions:**

This study suggests that LVI in histopathology is associated with a higher risk of BCR and advanced clinicopathological features in PCa patients and could serve as a poor prognostic factor in patients who underwent RP.

## Abstract 2

Supplemental Digital Content is available in the text

## 1 Introduction

Prostate cancer (PCa) is the second most prevalent cancer in people aged ≥50 years and poses a substantial burden on the healthcare system all over the world.^[1]^ With superior cancer control and functional outcomes, radical prostatectomy (RP) has become the gold standard treatment for localized PCa.^[2]^ However, approximately 40% of patients who undergo RP will experience biochemical recurrence (BCR),^[3]^ which is defined as an elevation in prostate-specific antigen (PSA) levels. BCR after RP is often assumed to represent clinical progression or distant metastases, indicating that the patients will need to be treated with secondary treatment.^[4,5]^

The traditional risk factors for BCR rely on known clinical and pathologic variables, including extraprostatic extension (EPE),^[6]^ seminal vesicle invasion (SVI),^[7]^ lymph node metastases (LNM)^[8]^ and positive surgical margin (PSM).^[9]^ However, the outcomes of surgically treated patients with adverse local pathologic features are not invariably poor,^[10]^ as not every patient suffers eventual cancer recurrence, and the consistent use of adjuvant radiotherapy could lead to considerable overtreatment. Consequently, research on the identification and evaluation of new prognostic predictors could help urologists precisely assess PCa risk, recurrence, and prognosis in the clinic.

Lymphovascular invasion (LVI) is defined as the presence of tumor cells in an endothelium-lined space. According to the International Society of Urological Pathology (ISUP) recommendation, LVI is part of the standard examination of RP specimens,^[11]^ and the reported incidence rates of LVI differ widely from 5% to 53% in patients who have undergone RP.^[12]^ Although there is general agreement that LVI is a significant predictor of BCR in univariate analyses of RP samples, not all studies have found LVI to be independently significant in multivariate analyses.^[13–15]^

Therefore, to further clarify the prognostic and clinicopathological value of LVI in PCa, we performed this meta-analysis based on published studies to evaluate whether the presence of LVI has a prognostic impact on BCR both in univariate and multivariate analyses.

## 2 Materials and methods

### 2.1 Literature search

This study was carried out in accordance with the guidelines of the Preferred Reporting Items for Systematic Reviews and Meta-Analyses (PRISMA).^[16]^ A comprehensive online search of the literature in the PubMed, EMBASE, and Web of Science databases up to June, 2018 was performed using the following keywords

(“prostate cancer” or “prostate and neoplasms”) and (“radical prostatectomy”) and (“lymphovascular invasion”) and (“biochemical recurrence” or “biochemical failure”). In addition, we checked potentially relevant publications by examining the reference lists in the recent reviews, meta-analyses, and cited articles to identify related articles. Only publications written in English with available full text were included in this meta-analysis. Because the studies included in this meta-analysis have been published, no ethical approval was required.

### 2.2 Inclusion and exclusion criteria

Studies included in the meta-analysis must meet all of the following criteria:

(1) articles published as full papers in English;
(2) all patients were diagnosed with PCa, and LVI was assessed by pathologists;
(3) studies excluded patients who received RP treatment;
(4) BCR after RP was defined in all studies; and
(5) the association between LVI and BCR was reported, and sufficient published data were available for estimating hazard ratios (HRs) from univariate or multivariate analyses with 95% confidence intervals (CIs).

Accordingly, the exclusion criteria were as follows:

(1) reviews, letters, case reports, editorials, and author responses;
(2) studies without sufficient data;
(3) studies that did not analyze the correlation between LVI and the BCR rate of PCa; and
(4) articles that contained elements that were inconsistent with the inclusion criteria.

If more than 1 article from the same cohort was identified, only the most recent and informative 1 was included.

### 2.3 Data extraction and quality assessment

Data were independently abstracted by 2 investigators (Zhenlei Zha and Hu Zhao) using a standard protocol and data collection form in accordance with PRISMA. Any controversy was resolved by discussion with and rereading by the third investigator (Bin Wu). The following data were extracted from the included studies: the first author's name, publication year and country, recruitment period, sample size, age of patients, preoperative PSA level, Gleason score (GS), pathological staging, definition of LVI and BCR, the number of patients with LVI and BCR, median time to follow-up, and the HRs of LVI in univariate and multivariate Cox analyses.

The quality of the eligible studies was evaluated according to the Newcastle–Ottawa Scale (NOS)^[17]^ guidelines, which contains 3 main areas:

(1) selection of the study population;
(2) comparability of the groups; and
(3) ascertainment of the outcome.

The total score ranges from 0 to 9, and the studies with scores of 6 or more were deemed of high quality, whereas scores of 0 to 5 were considered to indicate poor quality.

### 2.4 Statistical analyses

Stata 12.0 software (Stat Corp, College Station, TX) was used to perform the meta-analysis. The estimated effects of the LVI and BCR risk were calculated using HRs and 95% CIs. Heterogeneity was analysed by the Chi-square-based Q test and **I**^2^. *P* <.10 or **I**^2^ >50% was considered statistically significant heterogeneity. A fixed model (FE) and random effect model (RE) were used according to the **I**^2^ value of heterogeneity. Sensitivity analysis was used to validate the reliability of the outcomes via the sequential omission of individual studies from the meta-analysis. Subgroup analysis was performed to check whether the heterogeneity was influenced by the geographical region, date of publication, mean age, sample size, mean preoperative PSA (p-PSA), median follow-up or the different cutoff values for BCR. Funnel plots and Egger linear regression were used to explore whether any publication bias existed. Statistical significance was defined as *P* <.05 in a 2-tailed test.

To determine the significance of LVI in pathological diagnosis, we also studied the associations between LVI and the clinicopathological features of PCa. Dichotomous variables were calculated by odds ratios (ORs) and pooled OR with 95% CI. Information about EPE (yes vs no), pathological GS (≥7 vs <7), LNM (yes vs no), pathological stage (≥T3 vs <T2), surgical margin (positive vs negative) and SVI (yes vs no) were dichotomized. The event numbers were obtained from the original studies, and the ORs and 95% CIs were calculated.

## 3 Results

### 3.1 Literature search and study characteristics

**Figure 1:**
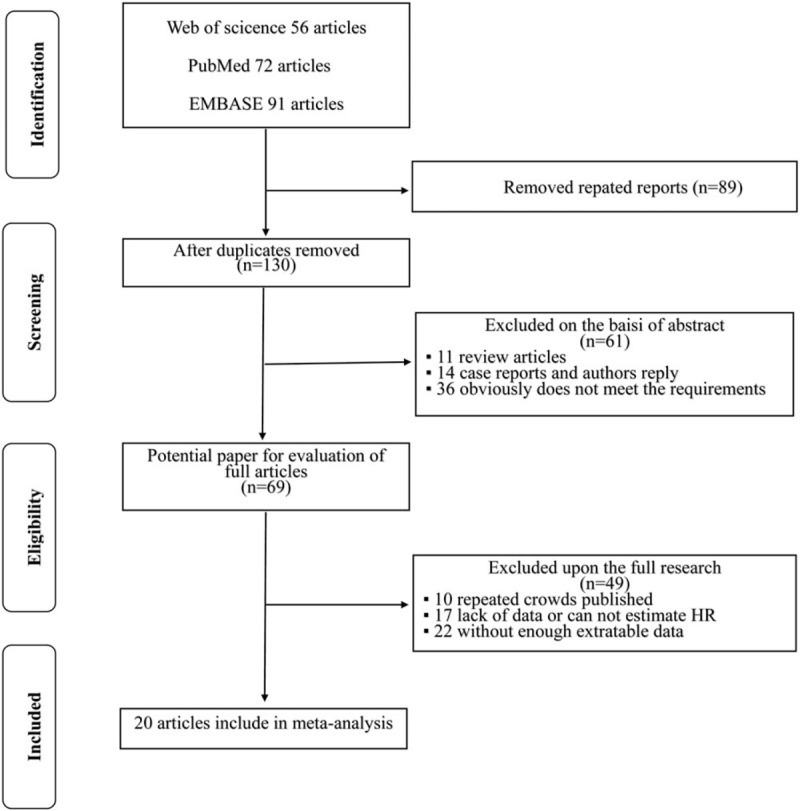
Flow diagram of the literature search and selection process.

The study search process used in this study is shown in Figure 1. A total of 219 potentially relevant studies were identified through systematic literature searches. After title and/or abstract screening, 150 studies were excluded because they were duplicates, reviews, case reports, or association between LVI and BCR was not evaluated. After the remaining studies (n = 69) were reviewed, 49 studies were excluded by the inclusion criteria: 39 due to the absence of HRs and/or enough extractable data, and 10 were repeated cohort publications. Finally, 20 retrospective studies^[9,13–15,18–33]^ involving 25,570 patients were included in the study.

The main characteristics and clinicopathological outcomes in the included studies are summarized in Tables 1 and 2. All studies were published between 2004 and 2017, of which 10 studies were conducted in Asia, 6 in North America, 2 in Germany, and 2 in multiple centers. The median or mean follow-up of patients ranged from 18.4 to 69.8 years. In regard to the prognostic value of LVI in PCa, 3 articles only reported univariate analysis, 6 articles only reported multivariate analysis, and 11 both reported univariate and multivariate analyses. The incidence of BCR after RP ranged from 7.6% to 34.5% in the studies. The cutoff value for BCR in these included studies was slightly different, with 17 studies using 0.2 ng.mL-1, 2 studies using 0.1 ng.mL-1, and 1 study using 0.4 ng.mL-1. The results of the methodological assessment by NOS ranged from scores of 7 to 9, indicating that all of the studies in our meta-analysis had high levels of methodological quality. (Supplementary Table S1)

**Table 1:**
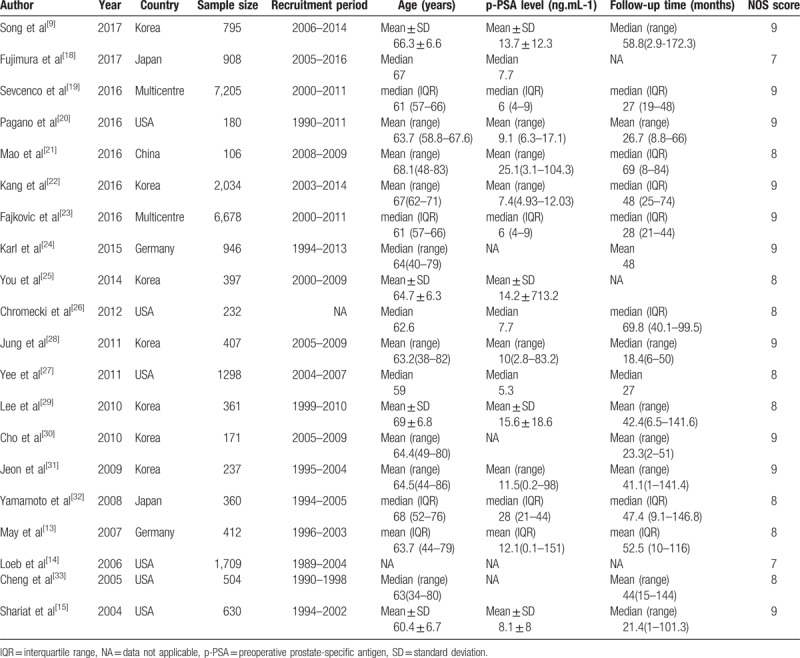
Main characteristics of the eligible studies.

**Table 2:**
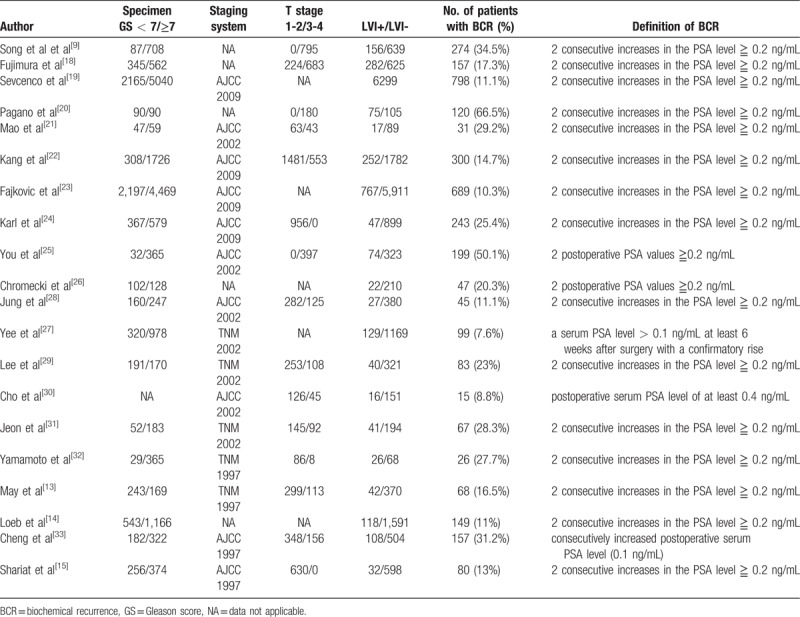
Tumour characteristics of the eligible studies.

### 3.2 Meta-analysis results

**Figure 2:**
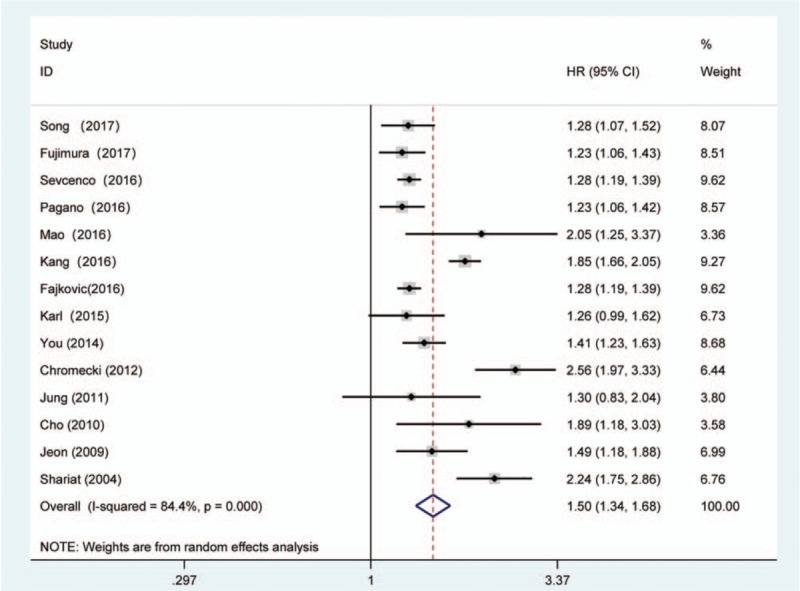
Forest plot and meta-analysis of studies evaluating the association between LVI and the BCR risk among men who underwent radical prostatectomy in univariate analysis mode. BCR = biochemical recurrence, LVI = lymphovascular invasion.

**Figure 3:**
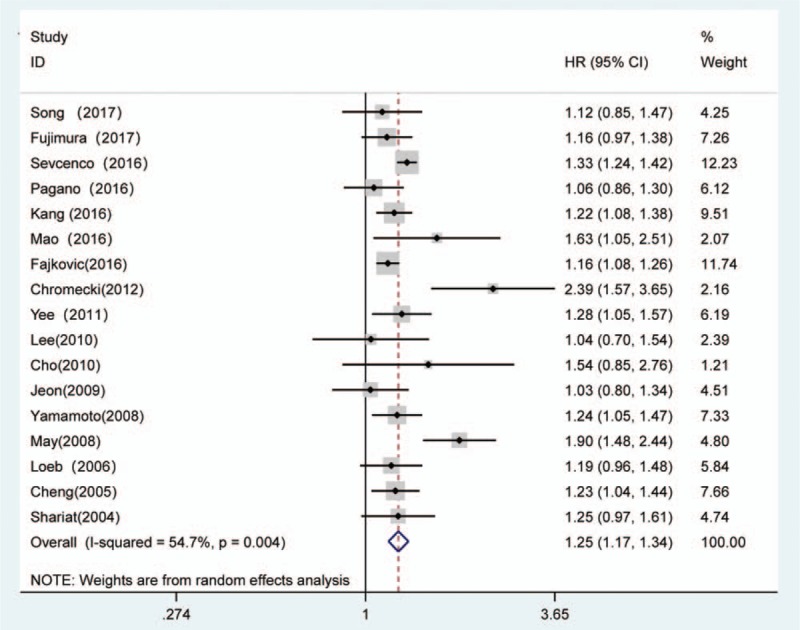
Forest plot and meta-analysis of studies evaluating the association between LVI and the BCR risk among men who underwent radical prostatectomy in multivariate analysis mode. BCR = biochemical recurrence, LVI = lymphovascular invasion.

The forest plots of the meta-analysis in our study demonstrated that LVI was associated with a higher BCR risk in univariate (RE model, pooled HR = 1.50, 95% CI: 1.34–1.68, *P* <.001, Fig. 2) and multivariate (RE model, pooled HR = 1.25, 95% CI: 1.17, 1.34, *P* <.001, Fig. 3) analyses. Due to the heterogeneity, we performed the subgroup analyses presented in Table 3 by stratifying the combined data according to the region (Asia vs No-Asia/Multicentre), publication year (≥2015 vs <2015), mean patient age (≥65 vs <65), sample size (≥500 vs <500), mean p-PSA level (≥10 vs <10), median follow-up time (≥30 vs <30) and the cutoff value for BCR (0.2 ng.mL-1 vs 0.1 ng.mL-1 or 0.4 ng.mL-1). The results showed that significant association between LVI and BCR based on Multicentre and mean p-PSA levels ≥10 ng.mL-1 in the univariate analysis. In the multivariate analysis, the subgroup analysis showed a significant association with BCR based on Asia region, mean age ≥65, sample size ≥500, mean p-PSA levels <10 ng.mL-1 and 0.1 ng.mL-1 or 0.4 ng.mL-1 cut-off values.

**Table 3:**
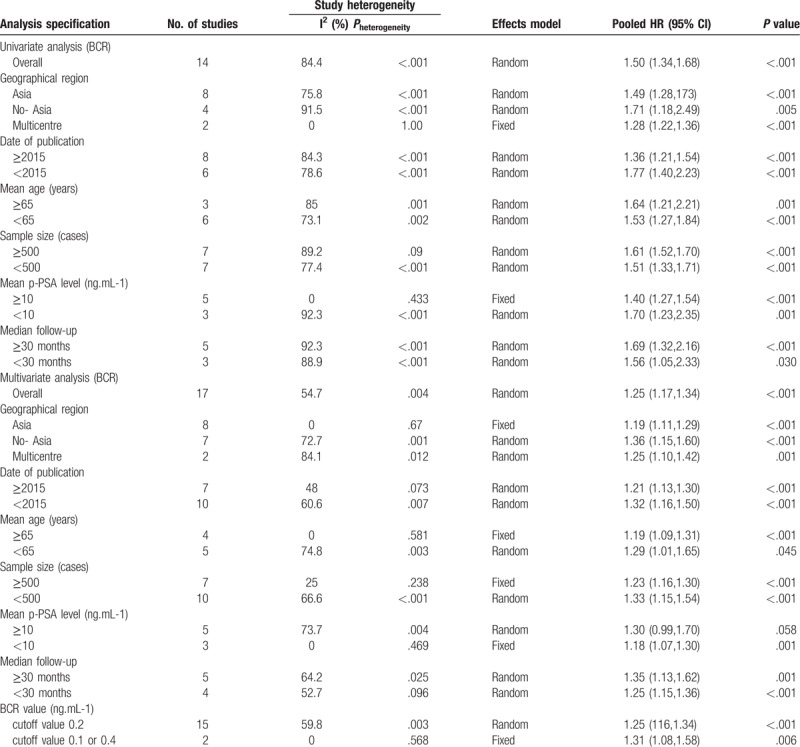
Summary and subgroup analyses for the eligible studies.

As shown in Table 4, patients with LVI were at a higher risk of having EPE (yes vs no: OR = 4.23, 95% CI: 1.86-9.61, *P* <.001, Supplementary Figure S1A), pathological GS (≥7 vs <7: OR = 5.46, 95% CI: 2.25–13.27, *P* <.001, Supplementary Figure S1B), LNM (yes vs no: OR = 18.56, 95% CI: 7.82–44.06, *P* <.001, Supplementary Figure S1C), higher pathological stage (≥ T3 vs <T2: OR = 6.75, 95% CI: 5.46–8.36, *P* <.001, Supplementary Figure S1D), PSM (positive vs negative: OR = 2.42, 95% CI: 1.57–3.72, *P* <.001, Supplementary Figure S1E) and SVI (yes vs no: OR = 5.72, 95% CI: 2.45–13.36, *P* <.001, Supplementary Figure S1F). Some significant interstudy heterogeneity was observed in EPE, pathological GS, LNM, PSM, and SVI but analyses of pathological stage did not exhibit significant heterogeneity.

**Table 4:**
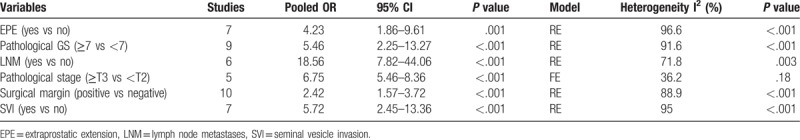
Meta-analysis of the associations between LVI and the clinicopathological features of PCa patients.

### 3.3 The sensitivity analysis and publication bias

**Figure 4:**
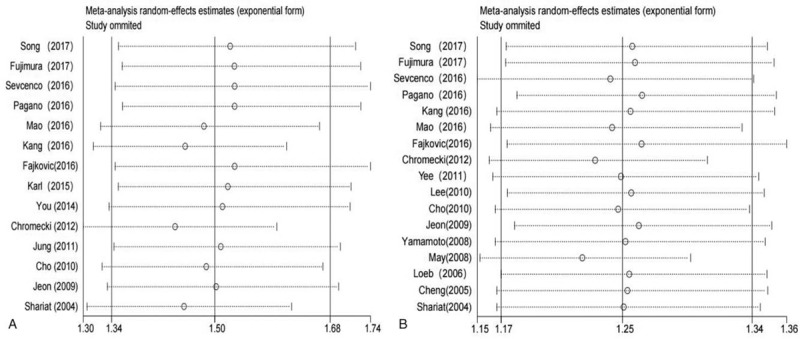
Sensitivity analysis of the association between LVI and the BCR risk in PCa patients. (A) Univariate analysis mode and (B) multivariate analysis mode. BCR = biochemical recurrence, LVI = lymphovascular invasion, PCa = prostate cancer.

**Figure 5:**
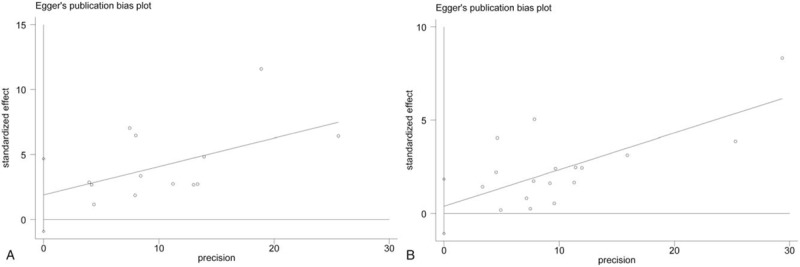
Funnel plots and Egger's tests for the evaluation of potential publication bias. (A) Univariate analysis mode and (B) multivariate analysis mode.

The overall significance did not change when any single study was omitted. Sensitivity analysis showed that the pooled HR for BCR ranged from 1.44 (95% CI, 1.30–1.59) to 1.53 (95% CI, 1.35–1.72) (Fig. 4A) in univariate analysis and from 1.22 (95% CI, 1.15–1.30) to 1.26 (95% CI, 1.17–1.35) (Fig. 4B) in multivariate analysis. These results indicated that the findings were reliable and robust. The funnel plots of the studies were symmetrical, and Egger's linear regression was performed. No significant publication bias was detected between these studies by univariate (p-Egger = 0.167, Fig. 5A) or multivariate analysis (p-Egger = 0.583, Fig. 5B).

## 4 Discussion

In the current treatment paradigm, BCR after RP serves as a trigger point for further treatment,^[34]^ and identifying effective predictors of BCR after the surgical operation to determine whether treatment is required is a main challenge in PCa research. Patients at high risk of BCR after RP can be offered adjuvant radiation therapy or androgen deprivation treatment.^[35]^ Early risk stratification for BCR among the heterogeneous patients undergoing RP could help physicians select patients who are more likely to benefit from adjuvant multimodal therapy. Several nomograms for prognostication of BCR after RP have been proposed.^[9,20,30]^ However, their validated prognostic accuracies are not yet optimal. A novel biomarker may provide a better understanding of an individual's tumor and improve the risk stratification of the patient population treated with RP.

Tumour metastasis is a complex process in which cancer cells obtain the ability to leave the primary tumor site via the lymphatic system and/or the bloodstream.^[36]^ LVI, as a detailed pathological finding, has been identified as an independent predictor of disease recurrence after curative treatment in multiple cancer types, including bladder cancer,^[37]^ gastric cancer,^[38]^ colorectal cancer,^[39]^ and PCa. Some authors suggest that the presence of LVI in PCa is associated with adverse oncological outcomes and higher recurrence rates,^[23]^ whereas others argue that LVI is not an independent predictor for prognosis.^[14,15,28]^ Ng et al^[40]^ suggested that there is insufficient evidence to recommend the routine use of LVI for clinical prognostication in a review article. A possible reason for the differences may arise from study design, sample size, source of the controls, or geographical region. All of these factors contribute to the limited statistical power in the published studies.

In 2016, Huang et al^[41]^ attempted to explore the impact of LVI on the BCR-free probability in a meta-analysis. They concluded that LVI may a predictor of the BCR–free probability in PCa patients. However, given the confused definition of LVI in the study by Huang et al, the conclusion of the study was not based on strong statistical evidence. In addition, the calculation method for pooled HRs and 95% CIs in the study by Huang et al was inappropriate. Compared to the results of a univariate analysis, the data from a multivariate analysis is more accurate, as it accounts for confounding factors.^[42]^ Therefore, it is inappropriate to put the data, which were extracted from 2 different analysis models, in a single forest plot in the meta-analysis. In addition, our study presented more studies in comparison with the study by Huang et al. As the search time reported in the meta-analyses from Huang et al ended in 2014, we added 9 extra studies with high quality from 2014 to 2017, thus providing more exact data evaluation for the pooled HRs and enabling more subgroup analyses. In addition, as we included more studies assessing the associations between LVI and the risk of BCR, our meta-analysis provides more reliable conclusions that reveal real associations compared with the study by Huang et al.

In the present study, among the 25,570 patients with PCa after RP, BCR was identified in 3647 (14.3%) patients. This meta-analysis supports that LVI was a strong independent predictor of BCR both in univariate (pooled HR = 1.50, *P* <.001) and multivariate (pooled HR = 1.25, *P* <.001) analyses. In the multivariate analysis, the subgroup analyses suggested that the associations were significant in the subgroups with an Asia region, mean age ≥65, sample size ≥500, mean p-PSA levels < 10 ng.mL-1 and 0.1 ng.mL-1 or 0.4 ng.mL-1 cut-off values. Besides, the association was also present in the subgroup with Multicentre and mean p-PSA levels ≥10 ng.mL-1 in univariate analysis. In addition, our results also suggested that PCa patients with LVI were likely to have a higher GS and pathological stage, PSMs, EPE, SVI, and LNM. The correlation between LVI and these factors revealed that LVI has the potential to be adopted as a dichotomous biomarker. The sensitivity analyses indicated that the findings were reliable and robust. In addition, there was no evidence of significant publication bias in these analyses according to Egger linear regression. Taken together, the current evidence suggests that LVI plays a pivotal role in cancer progression.

As a meta-analysis, the present study allows us to obtain a better understanding of the clinicopathological role of LVI in PCa patients. However, certain limitations in the meta-analysis should draw our attention as well. The first of which is its retrospective nature, despite the use of a large sample size. Second, we only included published studies written in English, which may cause selection bias. Third, although uniform criteria were used to select eligible studies, inherent differences among the studies still existed. Fourth, substantial heterogeneity was observed in the meta-analysis, and the heterogeneity was probably caused by differences in factors, such as the characteristics of the patients and variation in the cutoff values for BCR. Therefore, we should design randomized, controlled studies to provide more evidence of the prognostic importance of LVI in PCa patients.

## 5 Conclusions

In summary, although certain limitations exist, the results of the present study provide strong evidence that LVI was associated with a more aggressive tumor phenotype and could be regarded as a poor prognosis indicator for BCR in patients with PCa. These findings indicated that LVI expression is a potentially novel clinical prognostic factor in identifying individuals at an increased risk for BCR progression.

## Author contributions

**Conceptualization:** Wei Jiang, Lijin Zhang, Bin Wu.

**Data analysis:** Jun Yuan, Yuefang Jiang.

**Data curation:** Lijin Zhang, Zhenlei Zha, Hu Zhao, Yuan Jun.

**Literature search:** Zhenlei Zha, Hu Zhao, Bin Wu.

**Software:** Yuefang Jiang.

**Writing – original draft:** Wei Jiang, Lijin Zhang.

**Writing – review & editing:** Bin Wu, Lijin Zhang.

all authors approved the final manuscript.

## Supplementary Material

Supplemental Digital Content
