# Supplementary material for: The impact of lymphovascular invasion in patients with prostate cancer following radical prostatectomy and its association with their clinicopathological features: An updated PRISMA-compliant systematic review and meta-analysis
Source: Medicine (Baltimore). 2018 Dec 10;97(49):e13537. doi: 10.1097/MD.0000000000013537 (PMC6310490; doi:10.1097/MD.0000000000013537)

**Supplementary Table S1. Quality assessment of cohort studies included in this meta- analysis**

| **Study** | **Representativeness of the exposed cohort** | **Selection of the unexposed cohort** | **Ascertainment of exposure** | **Outcome of interest not present at start of study** | **Control for important factor or additional factor** | **Outcome assessment** | **Follow-up long enough for outcomes to occur** | **Adequacy of follow-up of cohort** | **Total quality scores** |
| --- | --- | --- | --- | --- | --- | --- | --- | --- | --- |
| Song et al.[^9^](#_ENREF_9) | ★ | ★ | ★ | ★ | ★★ | ★ | ★ | ★ | 9 |
| Fujimura et al.[^18^](#_ENREF_18) | ★ | ★ | ★ | ★ | ★ | ★ | ★ | — | 7 |
| Sevcenco et al.[^19^](#_ENREF_19) | ★ | ★ | ★ | ★ | ★★ | ★ | ★ | ★ | 9 |
| Pagano et al.[^20^](#_ENREF_20) | ★ | ★ | ★ | ★ | ★★ | ★ | ★ | ★ | 9 |
| Mao et al.[^21^](#_ENREF_21) | — | ★ | ★ | ★ | ★★ | ★ | ★ | ★ | 8 |
| Kang et al.[^22^](#_ENREF_22) | ★ | ★ | ★ | ★ | ★★ | ★ | ★ | ★ | 9 |
| Fajkovic et al.[^23^](#_ENREF_23) | ★ | ★ | ★ | ★ | ★★ | ★ | ★ | ★ | 9 |
| Karl et al.[^24^](#_ENREF_24) | ★ | ★ | ★ | ★ | ★★ | ★ | ★ | ★ | 9 |
| You et al.[^25^](#_ENREF_25) | ★ | ★ | ★ | ★ | ★★ | ★ | ★ | — | 8 |
| Chromecki et al.[^26^](#_ENREF_26) | ★ | ★ | ★ | ★ | ★ | ★ | ★ | ★ | 8 |
| Jung et al.[^28^](#_ENREF_28) | ★ | ★ | ★ | ★ | ★★ | ★ | ★ | ★ | 9 |
| Yee et al.[^27^](#_ENREF_27) | ★ | ★ | ★ | ★ | ★ | ★ | ★ | ★ | 8 |
| Lee et al.[^29^](#_ENREF_29) | ★ | ★ | ★ | ★ | ★ | ★ | ★ | ★ | 8 |
| Cho et al.[^30^](#_ENREF_30) | ★ | ★ | ★ | ★ | ★★ | ★ | ★ | ★ | 9 |
| Jeon et al.[^31^](#_ENREF_31) | ★ | ★ | ★ | ★ | ★★ | ★ | ★ | ★ | 9 |
| Yamamoto et al.[^32^](#_ENREF_32) | ★ | ★ | ★ | ★ | ★ | ★ | ★ | ★ | 8 |
| May et al.[^13^](#_ENREF_13) | ★ | ★ | ★ | ★ | ★ | ★ | ★ | ★ | 8 |
| Loeb et al.[^14^](#_ENREF_14) | ★ | ★ | ★ | ★ | ★ | ★ | ★ | — | 7 |
| Cheng et al.[^33^](#_ENREF_33) | ★ | ★ | ★ | ★ | ★ | ★ | ★ | ★ | 8 |
| Shariat et al.[^15^](#_ENREF_15) | ★ | ★ | ★ | ★ | ★★ | ★ | ★ | ★ | 9 |

Supplementary Figure 1. Forest plots of the association between LVI and the clinicopathological features of PCa patients: (S1A) EPE, (S1B) pathological GS, (S1C) LNM, (S1D) pathological stage, (S1E) surgical margin, and (S1F) SVI.


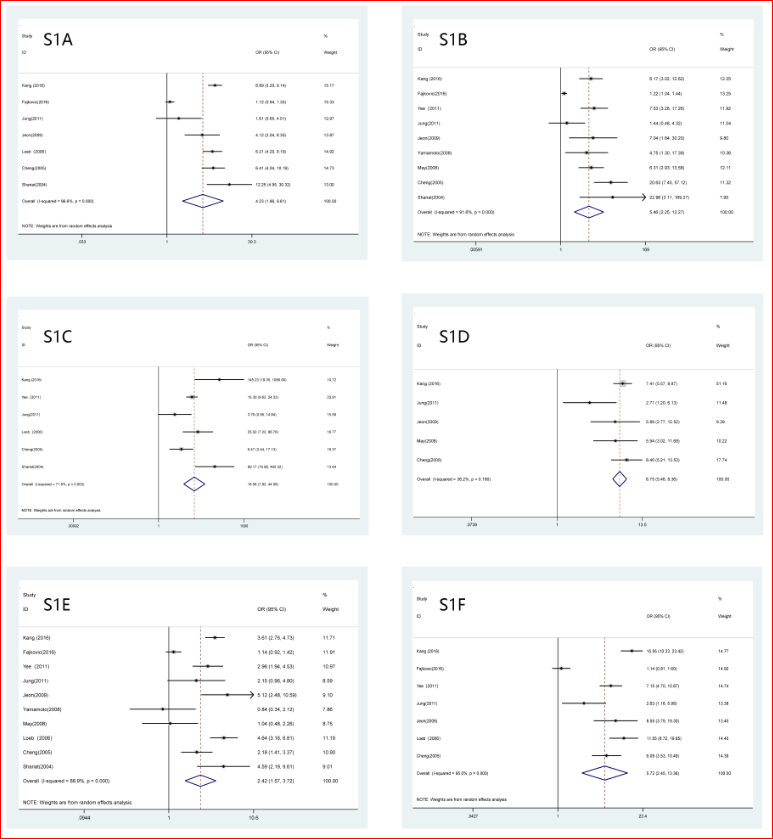

Supplement: Supplemental Digital Content [file medi-97-e13537-s001.doc]
